# Development, Implementation, and Evaluation of a Structured Teaching Module for Lumbar Puncture Training Among Medical Interns

**DOI:** 10.7759/cureus.114553

**Published:** 2026-08-14

**Authors:** Amit K Chauhan, Kanika Munish, Vidisha Vallabh, Anuradha Kusum, Shreesh Mehrotra, Divya Gupta

**Affiliations:** 1 Department of Anesthesiology, Himalayan Institute of Medical Sciences, Dehradun, IND; 2 Department of Pathology, Chhatrapati Shivaji Subharti Hospital, Netaji Subhash Chandra Bose Subharti Medical College, Meerut, IND; 3 Department of Community Medicine, Himalayan Institute of Medical Sciences, Dehradun, IND; 4 Department of Pathology, Himalayan Institute of Medical Sciences, Dehradun, IND

**Keywords:** competency-based medical education (cbme), doap, lumbar puncture, procedural competence, simulation-based medical education

## Abstract

Background

Lumbar puncture (LP) is an essential diagnostic and therapeutic bedside procedure. However, many medical interns have limited opportunities for supervised training before performing LP on patients, resulting in inadequate procedural competence and reduced confidence. Simulation-based education using the demonstration, observation, assistance, and performance (DOAP) approach offers a safe and effective method for teaching procedural skills. This study evaluated the effectiveness of a structured simulation-based teaching module in improving knowledge, confidence, procedural competence, and learner perceptions regarding lumbar puncture.

Methods

A pre- and post-intervention study was conducted in the Department of Anesthesiology and the Skills Laboratory Center at Himalayan Institute of Medical Sciences, Swami Rama Himalayan University, Dehradun, India, between January 1, 2025, and June 30, 2025. A total of 33 participants, including Bachelor of Medicine and Bachelor of Surgery (MBBS) medical interns, were enrolled. Participants underwent a structured teaching module using the DOAP method consisting of an interactive lecture, mannequin-based demonstration, supervised hands-on practice, and faculty feedback. Outcomes were assessed before and immediately after training using the investigator-developed Lumbar Puncture Training Assessment Questionnaire (LPTAQ), which evaluated knowledge, confidence, procedural competence, and post-training perceptions. Paired categorical responses were analyzed using the exact McNemar test.

Results

The mean age of participants was 24.15±1.44 years, and 57.6% were male. Significant improvements were observed in knowledge regarding termination of the adult spinal cord (54.5% vs. 84.8%, p=0.002), anatomical landmarks (69.7% vs. 93.9%, p=0.008), preferred vertebral level (57.6% vs. 93.9%, p<0.001), and indications for lumbar puncture (9.1% vs. 87.9%, p<0.001). Participants expressing confidence in performing lumbar puncture increased from 24.2% before training to 90.9% after training (p<0.001). Knowledge regarding the role of the stylet improved significantly (57.6% vs. 84.8%, p=0.004), while self-reported adequate procedural training increased from 9.1% to 90.9% (p<0.001). Confidence in identifying anatomical landmarks also improved significantly (63.6% vs. 93.9%, p=0.002). Significant gains were observed in knowledge related to post-procedure care, first-line management of post-lumbar puncture headache, and recognition of infection following LP. Overall, 90.9% of participants agreed that the structured teaching module improved their practical skills, 90.9% found mannequin-based demonstration helpful, 90.9% reported that the DOAP method improved procedural technique, and 93.9% felt confident performing lumbar puncture on real patients after training.

Conclusions

The structured simulation-based teaching module using the DOAP approach significantly improved knowledge, confidence, and self-reported procedural competence, as well as learner perceptions of lumbar puncture among medical interns. Incorporating structured simulation-based LP training into undergraduate and postgraduate medical education may enhance procedural preparedness, support competency-based medical education, and improve patient safety before interns perform lumbar puncture in clinical practice.

## Introduction

Lumbar puncture (LP) is one of the most frequently performed bedside procedures in clinical practice and serves an essential role in the diagnosis and management of a wide range of neurological and infectious diseases. It is performed for both diagnostic and therapeutic purposes across multiple medical disciplines [1]. Although evidence-based recommendations for procedural training are well established, their implementation in routine clinical practice remains inconsistent because of perceived logistical and educational barriers [2]. The competence of the operator has a direct impact on procedural success and patient safety, with higher levels of technical skill being associated with fewer complications and better clinical outcomes [3,4]. Despite undergoing clinical training, many junior doctors continue to experience uncertainty and lack confidence in performing LP. Several studies have reported that the knowledge and procedural skills of interns and residents often remain below expected competency levels, indicating gaps in conventional training methods [5-7]. Furthermore, novice operators experience significantly greater stress before and during LP compared with individuals who have previously performed the procedure, which may further compromise procedural performance [8].

Traditionally, procedural skills such as LP have been taught using the apprenticeship model commonly described as "see one, do one, teach one," in which interns first observe experienced clinicians before independently attempting the procedure [7]. While this approach has been widely adopted for decades, it offers limited opportunities for structured practice, standardized assessment, or supervised skill acquisition before patient contact. Consequently, considerable variation exists in LP techniques and practices, contributing to inconsistent procedural success and varying rates of complications [6]. These limitations have highlighted the need for structured educational strategies that enable interns to develop procedural competence in a controlled and supervised environment.

Simulation-based medical education has emerged as an effective strategy for teaching invasive procedures by providing realistic, risk-free opportunities for repeated practice and feedback [9]. Simulation-based training has been shown to be superior to traditional teaching approaches for invasive procedures and has demonstrated particular value in LP training by improving technical skills, increasing learner confidence, reducing anxiety, and enhancing patient safety [10]. Nevertheless, practical training in complex multistep bedside procedures such as LP continues to receive relatively limited emphasis during undergraduate and early postgraduate medical education, despite evidence that structured experiential learning improves procedural competence [11]. Structured simulation-based training may improve procedural preparedness by enhancing anatomical understanding, increasing first-attempt success, reducing the number of needle insertion attempts, and potentially improving patient safety before interns perform lumbar puncture in clinical practice [12].

In India, the transition from traditional knowledge-based education to Competency-Based Medical Education (CBME) has emphasized the need for systematic training and assessment of clinical skills. CBME was introduced in India in 2019 under the Medical Council of India (MCI), which was subsequently replaced by the National Medical Commission (NMC) in 2020 [13,14]. To facilitate effective implementation of the curriculum, the NMC established Nodal and Regional Centers responsible for faculty development and dissemination of CBME principles across medical colleges [15]. Program evaluation is an integral component of faculty development by ensuring that the desired knowledge, skills, and professional attitudes are effectively acquired and translated into educational practice [16,17].

However, successful transfer of educational interventions into routine teaching remains a complex process influenced by multiple institutional and learner-related factors [18]. Within the CBME framework, procedural skills are expected to be acquired through structured, supervised, and competency-based training rather than opportunistic learning during routine clinical practice. Simulation-based teaching using the demonstration, observation, assistance, and performance (DOAP) approach aligns well with CBME principles by providing standardized instruction, deliberate practice, formative feedback, and competency-focused assessment before patient exposure. Structured simulation-based teaching modules such as DOAP provide an effective educational strategy for achieving these competency-based learning objectives and can facilitate standardized procedural skills training before patient exposure [2,19].

Despite these educational reforms, structured LP training before direct patient exposure remains limited in many Indian medical institutions. As a result, interns often acquire procedural skills directly on patients, potentially increasing patient discomfort and the risk of avoidable complications. Recognizing this gap, the present study was undertaken to develop and evaluate a structured teaching module based on simulation and the DOAP approach. This study aimed to assess its effectiveness in improving the knowledge, confidence, self-reported procedural competence, and learner perceptions of medical interns before performing lumbar puncture in real clinical settings.

## Materials and methods

Study design and setting

This pre- and post-intervention study was conducted in the Department of Anesthesiology and the Skills Laboratory Center at Himalayan Institute of Medical Sciences (HIMS), Swami Rama Himalayan University, Dehradun, India, from January 1, 2025, to June 30, 2025. This study was undertaken to evaluate the effectiveness of a structured teaching module in improving participants' knowledge, confidence, and procedural competence regarding lumbar puncture.

Study participants

During the study period, all eligible MBBS medical interns posted to the skills laboratory were invited to participate. A total of 33 eligible interns were available, and all consented to participate, resulting in a final sample size of 33. Since the study was conducted as an educational intervention within a fixed training batch, universal sampling was adopted, and no formal sample size calculation was performed. Participation was voluntary, and written informed consent was obtained from all participants. All enrolled participants completed both the pre-training and post-training assessments.

Development of the structured teaching module

The structured teaching module was developed by faculty members from the Department of Anesthesiology based on standard anesthesiology textbooks, published literature on lumbar puncture, simulation-based medical education principles, and the competency-based medical education (CBME) curriculum recommended by the National Medical Commission (NMC), India. The content included indications, contraindications, relevant anatomy, patient preparation, informed consent, procedural steps, aseptic precautions, post-procedure care, recognition of complications, and safe clinical practice. The module was reviewed by experienced faculty members before implementation to ensure content validity and educational relevance.

Structured teaching module

The structured teaching module was delivered in the institutional skills laboratory using the DOAP approach. The training began with an interactive lecture covering the indications, contraindications, relevant spinal anatomy, equipment required, patient positioning, aseptic precautions, procedural steps, potential complications, and post-procedure care related to lumbar puncture. This was followed by a faculty demonstration of the lumbar puncture procedure on a mannequin simulator. Subsequently, each participant performed the procedure on the lumbar puncture mannequin under direct faculty supervision. Immediate individualized feedback was provided throughout the practical session to reinforce correct technique and correct procedural errors. Participants were encouraged to repeat the procedure until they became familiar with each procedural step and demonstrated satisfactory understanding of the technique before completing the training. The entire training session, including the lecture, demonstration, supervised hands-on practice, feedback, and assessment, lasted approximately 2-3 h. The final assessment was conducted exclusively on a lumbar puncture mannequin under direct faculty supervision. No participant performed lumbar puncture on actual patients as part of the study. Consequently, objective procedural performance, successful lumbar puncture on patients, and patient-based procedural outcomes were not evaluated.

Data collection

Data were collected before and immediately after the structured teaching module using the Lumbar Puncture Training Assessment Questionnaire (LPTAQ). The questionnaire assessed participants' knowledge, confidence, self-reported procedural competence, and perceptions regarding lumbar puncture training. Knowledge was assessed using multiple-choice questions covering anatomy, indications, procedural technique, post-procedure care, and complication management. Confidence, self-reported procedural competence, and learner perceptions were assessed using structured questionnaire items with predefined response options. For statistical analysis, responses were dichotomized into correct/incorrect for knowledge items and positive/negative for confidence, procedural competence, and perception domains, as described in the appendix.

Lumbar Puncture Training Assessment Questionnaire (LPTAQ)

The LPTAQ is a structured investigator-developed questionnaire designed to assess learning outcomes following lumbar puncture training. It was developed after a review of standard anesthesiology textbooks, published literature, and established recommendations on lumbar puncture. The draft questionnaire was reviewed by three faculty members from the Department of Anesthesiology with expertise in medical education and procedural skills to evaluate its content relevance, clarity, and comprehensiveness. Based on their recommendations, appropriate modifications were made before pilot testing. The revised questionnaire was pilot tested among five participants who were not included in the final study to assess readability, comprehension, and completion time. Feedback obtained during pilot testing was incorporated into the final version.

Formal psychometric evaluation of the questionnaire, including assessment of the content validity index, internal consistency, inter-rater reliability, and test-retest reliability, was not performed. The questionnaire was developed specifically for this educational intervention and underwent expert review and pilot testing to establish content relevance, clarity, and feasibility before implementation.

The final questionnaire is provided in the appendix covering baseline characteristics; knowledge of lumbar puncture anatomy, indications, and procedural steps; confidence in performing lumbar puncture; perceptions regarding the structured teaching module; skill-related knowledge, including needle insertion angle and the role of the stylet; self-reported procedural competence, including adequacy of training and confidence in identifying anatomical landmarks; and knowledge of post-procedure care, complication recognition, and patient safety.

Statistical analysis

Data were entered into Microsoft Excel (Redmond, WA: Microsoft Corp.) and analyzed using IBM SPSS Statistics version 27.0 (Armonk, NY: IBM Corp.). Continuous variables were expressed as mean±standard deviation (SD), whereas categorical variables were presented as frequency and percentage. Changes in paired categorical responses between the pre- and post-training assessments were analyzed using the exact McNemar test. Statistical significance was defined as a two-tailed p<0.05.

## Results

A total of 33 participants were included in the study. The mean age of the participants was 24.15±1.44 years. Among them, 19 (57.6%) were male, and 14 (42.4%) were female (Table 1). Knowledge regarding lumbar puncture improved after the structured teaching module. The proportion of participants who correctly identified the termination of the adult spinal cord increased from 18 (54.5%) before training to 28 (84.8%) after training (McNemar χ^2^=8.10, p=0.002). Correct identification of the anatomical landmark (iliac crest) improved from 23 (69.7%) to 31 (93.9%) (McNemar χ^2^=6.13, p=0.008). Correct identification of the vertebral level for lumbar puncture increased from 19 (57.6%) to 31 (93.9%) (McNemar χ^2^=10.08, p<0.001). Knowledge of the informed consent process showed only a slight increase from 29 (87.9%) to 30 (90.9%), which was not statistically significant (McNemar χ^2^=0.0, p=1.0). The greatest improvement was observed in identifying the indications for lumbar puncture, where correct responses increased from three (9.1%) before training to 29 (87.9%) after training (McNemar χ^2^=24.04, p<0.001) (Table 2).

**Table 1:** Baseline characteristics of the study participants (n=33). Data are presented as mean±standard deviation (SD) or number (%).

Characteristics	Value
Age (years), mean±SD	24.15±1.44
Male, n (%)	19 (57.6)
Female, n (%)	14 (42.4)

**Table 2:** Comparison of correct responses to knowledge-based questions before and after the structured teaching module. Data are presented as number (%). Paired categorical variables were compared using the exact McNemar test. McNemar χ² values are reported with continuity correction (cc). P<0.05 was considered statistically significant.

Questions	Before training, n (%)	After training, n (%)	McNemar χ^2^ (cc)	Exact p-Value
Correct identification of the termination of the adult spinal cord	18 (54.5)	28 (84.8)	8.10	0.002
Correct identification of the anatomical landmark for lumbar puncture (iliac crest)	23 (69.7)	31 (93.9)	6.13	0.008
Correct identification of the vertebral level for lumbar puncture (L3-L4/L4-L5)	19 (57.6)	31 (93.9)	10.08	<0.001
Correct understanding of the informed consent process before lumbar puncture	29 (87.9)	30 (90.9)	0.0	1.0
Correct identification of the indications for lumbar puncture	3 (9.1)	29 (87.9)	24.04	<0.001

Participants' confidence in performing lumbar puncture improved markedly after the structured teaching module. The proportion of participants expressing confidence increased from eight (24.2%) before training to 30 (90.9%) after training, and this improvement was statistically significant (McNemar χ^2^=20.05, p<0.001) (Table 3).

**Table 3:** Participants' confidence before and after structured lumbar puncture training. Data are presented as number (%). Paired categorical variables were analyzed using the exact McNemar test. McNemar χ² values are reported with continuity correction (cc). P<0.05 was considered statistically significant.

Outcome	Before training, n (%)	After training, n (%)	McNemar χ^2^ (cc)	Exact p-Value
Participants expressing confidence in performing lumbar puncture	8 (24.2)	30 (90.9)	20.05	<0.001

Most participants had a positive perception of the structured teaching module. A total of 30 (90.9%) participants agreed that the structured teaching module improved their practical skills, 30 (90.9%) found the mannequin-based demonstration to be very helpful, and 30 (90.9%) reported that the DOAP method improved their procedural technique. In addition, 31 (93.9%) participants felt confident performing lumbar puncture on real patients after completing the training (Table 4).

**Table 4:** Participants' perceptions of the structured teaching module. Data are presented as number (%). DOAP: demonstration, observation, assistance, and performance

Statements	Positive response, n (%)
Structured teaching module improved practical skills	30 (90.9)
Mannequin-based demonstration was very helpful	30 (90.9)
DOAP method improved procedural technique	30 (90.9)
Participants felt confident performing lumbar puncture on real patients after training	31 (93.9)

Skill-related knowledge improved after the structured teaching module. The proportion of participants who correctly identified the needle insertion angle increased from 26 (78.8%) before training to 29 (87.9%) after training; however, this improvement was not statistically significant (McNemar χ^2^=1.33, p=0.250). Correct identification of the primary role of the stylet increased from 19 (57.6%) before training to 28 (84.8%) after training, representing a statistically significant improvement (McNemar χ^2^=7.11, p=0.004) (Table 5).

**Table 5:** Comparison of correct responses to skill-related knowledge questions before and after the structured teaching module. Data are presented as number (%). Paired categorical variables were compared using the exact McNemar test. McNemar χ² values are reported with continuity correction (cc). Statistical significance was considered at p<0.05.

Questions	Before training, n (%)	After training, n (%)	McNemar χ² (cc)	Exact p-Value
Correct identification of the needle insertion angle (90° to the skin)	26 (78.8)	29 (87.9)	1.33	0.250
Correct identification of the primary role of the stylet	19 (57.6)	28 (84.8)	7.11	0.004

Self-reported procedural competence improved after the structured teaching module. The proportion of participants reporting adequate training in lumbar puncture procedures increased from three (9.1%) before training to 30 (90.9%) after training, showing a statistically significant improvement (McNemar χ^2^=24.04, p<0.001). Familiarity with the lumbar puncture checklist increased from 24 (72.7%) to 29 (87.9%), but this change was not statistically significant (McNemar χ^2^=3.20, p=0.063). Confidence in identifying anatomical landmarks improved from 21 (63.6%) before training to 31 (93.9%) after training, which was statistically significant (McNemar χ^2^=8.10, p=0.002) (Table 6).

**Table 6:** Self-reported procedural competence before and after the structured teaching module. Data are presented as number (%). Paired categorical variables were compared using the exact McNemar test. McNemar χ² values are reported with continuity correction (cc). P<0.05 was considered statistically significant.

Outcomes	Before training, n (%)	After training, n (%)	McNemar χ^2^ (cc)	Exact p-Value
Participants reporting adequate training in lumbar puncture procedures	3 (9.1)	30 (90.9)	24.04	<0.001
Participants familiar with the lumbar puncture checklist	24 (72.7)	29 (87.9)	3.20	0.063
Participants confident in identifying anatomical landmarks	21 (63.6)	31 (93.9)	8.10	0.002

Knowledge related to post-procedure care and safety improved after the structured teaching module. Correct identification of the recommended post-procedure care following lumbar puncture increased from five (15.2%) before training to 28 (84.8%) after training, showing a statistically significant improvement (McNemar χ^2^=21.04, p<0.001). Correct identification of common complications following lumbar puncture increased from 25 (75.8%) to 30 (90.9%), but this improvement was not statistically significant (McNemar χ^2^=3.20, p=0.063). Knowledge of the most common cause of post-lumbar puncture headache improved from 25 (75.8%) to 29 (87.9%), although the difference was not statistically significant (McNemar χ^2^=2.25, p=0.125). Correct identification of the appropriate first-line management of post-lumbar puncture headache increased from 23 (69.7%) before training to 29 (87.9%) after training, with a statistically significant improvement (McNemar χ^2^=4.17, p=0.031). Correct identification of a common sign of infection following lumbar puncture also improved significantly from five (15.2%) before training to 29 (87.9%) after training (McNemar χ^2^=22.04, p<0.001) (Table 7).

**Table 7:** Comparison of correct responses to post-procedure care and safety questions before and after the structured teaching module. Data are presented as number (%). Paired categorical variables were compared using the exact McNemar test. McNemar χ² values are reported with continuity correction (cc). P<0.05 was considered statistically significant.

Questions	Before training, n (%)	After training, n (%)	McNemar χ^2^ (cc)	Exact p-Value
Correct identification of recommended post-procedure care following lumbar puncture	5 (15.2)	28 (84.8)	21.04	<0.001
Correct identification of common complications following lumbar puncture	25 (75.8)	30 (90.9)	3.20	0.063
Correct identification of the most common cause of post-lumbar puncture headache	25 (75.8)	29 (87.9)	2.25	0.125
Correct identification of the appropriate first-line management of post-lumbar puncture headache	23 (69.7)	29 (87.9)	4.17	0.031
Correct identification of a common sign of infection following lumbar puncture	5 (15.2)	29 (87.9)	22.04	<0.001

## Discussion

The present study evaluated the effectiveness of a structured teaching module based on the DOAP approach in improving the knowledge, confidence, procedural competence, and perceptions of medical interns regarding lumbar puncture. The findings demonstrated significant improvements across multiple domains following the intervention, indicating that structured simulation-based training is an effective educational strategy for preparing novice learners before they perform lumbar puncture on patients.

Previous studies have demonstrated a clear association between operator competence and procedural complications, emphasizing the importance of adequate training before independent clinical practice [3,4]. Consistent with these observations, participants in the present study showed significant improvements in fundamental knowledge related to spinal anatomy, procedural indications, informed consent, needle insertion technique, and the role of the stylet following completion of the structured teaching module. In addition, participants reported greater confidence in identifying anatomical landmarks and performing the procedure, suggesting that the combination of didactic teaching, mannequin-based simulation, and supervised practice effectively enhanced both cognitive understanding and procedural readiness.

Earlier studies have also reported that novice operators experience higher levels of stress and anxiety before and during lumbar puncture compared with individuals who have previous procedural experience [8]. Although stress levels were not directly measured in the present study, the marked improvement in participants' self-reported confidence after training suggests that structured simulation-based education may help reduce apprehension associated with performing lumbar puncture. Improved confidence is particularly important during the early stages of clinical training because it enables learners to approach procedures in a more systematic and controlled manner while maintaining patient safety.

For many years, procedural training has relied on the traditional apprenticeship model of "see one, do one, teach one," in which interns learn primarily through observation followed by supervised practice on patients [7]. While this approach remains an important component of clinical education, it does not always provide standardized instruction or sufficient opportunities for deliberate practice before patient contact. The significant improvements observed in the present study support the incorporation of structured teaching modules using the DOAP approach and simulation-based learning. Participants demonstrated improved familiarity with the lumbar puncture checklist, enhanced understanding of procedural steps, and increased confidence in technical performance, highlighting the educational value of structured skills laboratory training before clinical application.

Simulation-based medical education has been recognized as an effective method for teaching invasive procedures by allowing learners to practice repeatedly in a safe environment without exposing patients to unnecessary risk [6,10]. The present findings are in agreement with these reports and demonstrate that simulation-based teaching can substantially improve learners' procedural competence and confidence. Such training provides immediate faculty feedback, reinforces correct technique, and allows learners to develop psychomotor skills before transitioning to real patient care.

Knowledge regarding post-procedure care and complication management is equally important for ensuring patient safety after lumbar puncture. Previous literature has highlighted deficiencies in learners' understanding of post-procedure care and complication recognition [11]. In the present study, participants demonstrated significant improvements in knowledge related to comprehensive post-procedure care, recognition of complications, and the management of post-lumbar puncture headache following the structured teaching module. Although improvements in identifying the common cause of post-lumbar puncture headache, first-line management, and recognition of infection were comparatively smaller, these findings likely reflect the relatively higher baseline knowledge observed in these areas before training rather than a lack of effectiveness of the educational intervention.

The strengths of this study include the use of a structured investigator-developed questionnaire, standardized delivery of the teaching module using the DOAP approach, and evaluation of multiple educational domains, including knowledge, confidence, self-reported procedural competence, and learner perceptions. However, several limitations should be acknowledged. The study was conducted at a single institution with a relatively small sample size, which may limit the generalizability of the findings. In addition, the study employed a single-group pre- and post-design without a control group. Therefore, the observed improvements cannot be attributed exclusively to the intervention, and the immediate post-intervention assessment may have introduced testing effects. Furthermore, the inclusion of medical interns may have resulted in heterogeneity in baseline procedural experience.

Procedural competence was assessed using self-reported measures and knowledge-based assessments rather than objective performance measures, such as an objective structured clinical examination (OSCE), a faculty-rated procedural checklist, a simulator-based competency score, or supervised clinical performance assessment. Therefore, the observed improvements reflect self-reported preparedness and knowledge acquisition rather than objectively demonstrated procedural competence. In addition, although the investigator-developed LPTAQ underwent expert review and pilot testing before implementation, formal psychometric validation, including assessment of the content validity index, internal consistency, inter-rater reliability, and test-retest reliability, was not performed. Therefore, the measurement properties of the questionnaire should be interpreted with appropriate caution. Finally, long-term retention of knowledge and procedural skills was not assessed because outcomes were evaluated immediately after completion of the teaching module. Consequently, the observed improvements may reflect short-term learning and do not establish sustained knowledge retention or maintenance of procedural competence. Future multicenter studies with larger sample sizes, objective skill assessments, supervised clinical performance evaluations, formal validation of assessment instruments, and longer follow-up are needed to determine whether simulation-based training translates into sustained clinical competence and improved patient outcomes. Reassessment after three or six months would further help determine long-term knowledge retention and maintenance of procedural skills.

## Conclusions

The structured teaching module significantly improved medical interns' knowledge, confidence, and self-reported procedural competence regarding lumbar puncture. Simulation-based training using the DOAP approach enhanced participants' understanding of procedural steps, anatomical landmarks, post-procedure care, and patient safety while increasing their confidence in performing the procedure. Participants also reported high satisfaction with the training and perceived it as beneficial for developing practical skills. Incorporating structured simulation-based lumbar puncture training into undergraduate medical education may facilitate competency-based learning, improve procedural preparedness, and promote safer clinical practice before patient exposure.

## Appendices

Coding scheme used for analysis of the questionnaire

**Table 8:** Baseline characteristics of study participants.

Variables	Response options
Age (years)	Continuous variable
Gender	Male/female

**Table 9:** Knowledge assessment of lumbar puncture.

Questions	Response options	Code
Where does the adult spinal cord usually terminate?	Lower border of L1 vertebra	1
	Upper border of L2 vertebra	1
	Lower border of L2 vertebra	0
	L3 vertebra	0
Which anatomical landmark is commonly used to identify the lumbar puncture site?	Iliac crest	1
	Sacral crest	0
	Spinous process of T12	0
	Coccyx	0
Which vertebral level is preferred for lumbar puncture?	L1-L2	0
	L2-L3	0
	L3-L4	1
	L4-L5	1
Before performing lumbar puncture, informed consent should be	Not required	0
	Verbal only	0
	Written after explaining the procedure, risks, benefits, and alternatives	1
	Obtained only from relatives	0
Which of the following is an indication for lumbar puncture?	Suspected meningitis	0
	Subarachnoid hemorrhage	0
	Diagnostic evaluation of neurological disorders	0
	All of the above	1

**Table 10:** Confidence assessment regarding lumbar puncture.

Question	Response options	Code
How confident are you in performing a lumbar puncture?	Not confident	0
	Slightly confident	0
	Moderately confident	1
	Very confident	1

**Table 11:** Perception of the structured teaching module (post-test only). DOAP: demonstration, observation, assistance, and performance

Statements	Response options	Code
The structured teaching module improved my practical skills	Strongly disagree	0
	Disagree	0
	Neutral	0
	Agree	1
	Strongly agree	1
The mannequin demonstration was very helpful	Strongly disagree	0
	Disagree	0
	Neutral	0
	Agree	1
	Strongly agree	1
The DOAP method improved my procedural technique	Strongly disagree	0
	Disagree	0
	Neutral	0
	Agree	1
	Strongly agree	1
I feel confident performing lumbar puncture on real patients after this training	Strongly disagree	0
	Disagree	0
	Neutral	0
	Agree	1
	Strongly agree	1

**Table 12:** Skill-related knowledge assessment.

Questions	Response options	Code
What is the correct angle for inserting the spinal needle?	10°-15°	0
	30°-45°	0
	60°	0
	90° to the skin	1
What is the primary role of the stylet?	Helps the needle penetrate the skin	0
	Maintains the patency of the needle during insertion	1
	Measures cerebrospinal fluid	0
	Provides local anesthesia	0

**Table 13:** Self-reported procedural competence.

Questions	Response options	Code
Have you received adequate training in lumbar puncture procedures?	No	0
	Theory only	0
	Practical training	1
	Practical training with supervision	1
How familiar are you with the lumbar puncture procedural checklist?	Not familiar	0
	Somewhat familiar	1
	Very familiar	1
Are you confident in identifying the correct anatomical landmarks?	No	0
	Yes, with guidance	0
	Yes, independently	1
	Yes, confidently	1

**Table 14:** Post-procedure care and safety assessment.

Questions	Response options	Code
Which of the following represents comprehensive post-procedure care following lumbar puncture?	Bed rest only	0
	Hydration only	0
	Monitoring vital signs only	0
	Bed rest, hydration, monitoring vital signs, and prevention of infection or complications	1
Which of the following are common complications following lumbar puncture?	Post-puncture headache	0
	Infection	0
	Nerve injury	0
	Bloody cerebrospinal fluid	0
	All of the above	1
What is the most common cause of post-lumbar puncture headache?	Low cerebrospinal fluid pressure	1
	Infection	0
	Spinal cord injury	0
	Nerve damage	0
What is the most appropriate first-line management of post-lumbar puncture headache?	Intravenous antibiotics	0
	Encourage adequate hydration and conservative management	1
	Repeat lumbar puncture	0
	Surgical intervention	0
Which finding is most suggestive of infection following lumbar puncture?	Severe headache with fever	1
	Vomiting without fever	0
	Decreased urine output	0
	Lower limb numbness	0

Note: For statistical analysis, responses coded as 1 were considered correct (knowledge domains) or positive (confidence, perception, and procedural competence domains). Responses coded as 0 were considered incorrect or negative. Binary coding was performed before statistical analysis to derive the outcomes presented in Tables 2-7.

## References

[1] Mishra B, Vishnu VY (2021). Lumbar puncture: indications, challenges and recent advances. Touch Rev Neurol.

[2] Motola I, Devine LA, Chung HS, Sullivan JE, Issenberg SB (2013). Simulation in healthcare education: a best evidence practical guide. AMEE guide no. 82. Med Teach.

[3] Reeder C, McClerking C, King TS, Browning K (2021). Improving bedside procedures through the implementation of case-based simulation and mastery learning for lumbar puncture training in novice advanced practice providers. J Nurse Pract.

[4] Boon JM, Abrahams PH, Meiring JH, Welch T (2004). Lumbar puncture: anatomical review of a clinical skill. Clin Anat.

[5] Henriksen MJ, Wienecke T, Thagesen H, Jacobsen RV, Subhi Y, Ringsted C, Konge L (2017). Assessment of residents readiness to perform lumbar puncture: a validation study. J Gen Intern Med.

[6] Gaubert S, Blet A, Dib F (2021). Positive effects of lumbar puncture simulation training for medical students in clinical practice. BMC Med Educ.

[7] McMillan HJ, Writer H, Moreau KA (2016). Lumbar puncture simulation in pediatric residency training: improving procedural competence and decreasing anxiety. BMC Med Educ.

[8] Vrillon A, Gonzales-Marabal L, Ceccaldi PF, Plaisance P, Desrentes E, Paquet C, Dumurgier J (2022). Using virtual reality in lumbar puncture training improves students learning experience. BMC Med Educ.

[9] Elendu C, Amaechi DC, Okatta AU, Amaechi EC, Elendu TC, Ezeh CP, Elendu ID (2024). The impact of simulation-based training in medical education: a review. Medicine (Baltimore).

[10] Muñoz-Leija D, González-Colmenero FD, Ramiréz-Mendoza DA, López-Cabrera NG, Llanes-Garza HA, Palacios-Ríos D, Negreros-Osuna AA (2024). Development and evaluation of an in-house lumbar puncture simulator for first-year resident lumbar puncture procedure learning. Cureus.

[11] von Cranach M, Backhaus T, Brich J (2019). Medical students' attitudes toward lumbar puncture - and how to change. Brain Behav.

[12] Stögbauer J, Jonas S, Hao W (2026). Simulator-based training sustainably improves confidence, theoretical knowledge, and success rates in lumbar puncture among medical students: a prospective case-control study. J Med Educ Curric Dev.

[13] Revi KS, Chaudhari GR, Kumar R, Khan SS (2025). The effect of competency based medical education on medical students. Int J Med Pharm Res.

[14] Telang A, Rathod S, Supe A, Nebhinani N, Mathai S (2017). Faculty views on competency-based medical education during mentoring and learning web sessions: an observational study. J Educ Technol Health Sci.

[15] Soundariya K, Kalaiselvan G, Rajalakshmi M, Sindhuri R (2022). Implementation and evaluation of competency-based medical education in phase I of undergraduate medical curriculum. J Adv Med Educ Prof.

[16] Shrivastava SR, Shrivastava PS (2023). Program evaluation in the field of medical education: need and approaches. Curr Med Issues.

[17] Kojuri J, Amini M, Karimian Z (2015). Needs assessment and evaluation of a short course to improve faculties teaching skills at a former World Health Organization regional teacher training center. J Adv Med Educ Prof.

[18] Yelon SL, Ford JK, Anderson WA (2014). Twelve tips for increasing transfer of training from faculty development programs. Med Teach.

[19] Chacko TV (2021). Giving feedback in the new CBME curriculum paradigm: principles, models and situations where feedback can be given. J Educ Technol Health Sci.

